# *Ureaplasma urealyticum* osteomyelitis of the greater trochanter in a patient with multiple sclerosis using ocrelizumab – a case report

**DOI:** 10.5194/jbji-9-167-2024

**Published:** 2024-06-13

**Authors:** Fred Ruythooren, Stijn Ghijselings, Melissa Depypere, Willem-Jan Metsemakers, Liesbet Henckaerts, Nathalie Noppe, Georges Vles

**Affiliations:** 1 Department of Orthopaedic Surgery, University Hospitals Leuven – Gasthuisberg, Leuven, Belgium; 2 Institute for Orthopaedic Research and Training (IORT), University Hospitals Leuven – Gasthuisberg, Leuven, Belgium; 3 Department of Laboratory Medicine, University Hospitals Leuven – Gasthuisberg, Leuven, Belgium; 4 Department of Microbiology, Immunology and Transplantation, Laboratory for Clinical Infectious and Inflammatory Disorders, KU Leuven, Leuven, Belgium; 5 Department of Trauma Surgery, University Hospitals Leuven – Gasthuisberg, Leuven, Belgium; 6 Department of Development and Regeneration, KU Leuven, Leuven, Belgium; 7 Department of General Internal Medicine, University Hospitals Leuven – Gasthuisberg, Leuven, Belgium; 8 Department of Radiology, University Hospitals Leuven – Gasthuisberg, Leuven, Belgium

## Abstract

Ocrelizumab – a monoclonal anti-CD20 antibody used in treatment of multiple sclerosis (MS) – marks significant progress in treating autoimmune diseases but raises susceptibility to opportunistic infections due to hypogammaglobulinemia. A young MS patient developed osteomyelitis from persistent *Ureaplasma urealyticum* urethritis, which was diagnosed with specialized polymerase chain reaction and resolved with targeted antibiotics. A multidisciplinary approach is crucial for managing such infections.

## 1 Introduction

The introduction of monoclonal antibodies has caused a revolution in the management of autoimmune diseases. These therapies tend to be associated with a range of adverse effects that are typically related to their effect on the immune system and immune cells. Ocrelizumab – a recombinant human anti-CD20 monoclonal antibody used in the treatment of multiple sclerosis (MS) – is typically associated with prolonged B-cell depletion and hypogammaglobulinemia that increases the risk for (mainly respiratory) bacterial or viral infections (Mikulska et al., 2018).

*Ureaplasma urealyticum* belongs to the Mycoplasmataceae family. The *Ureaplasma* genus hydrolyzes urea to generate adenosine triphosphate with ammonia as a byproduct. Ammonia increases pH and limits growth in culture (Huang and Shah, 2023a). *U. urealyticum* is considered one of the smallest known prokaryotic genomes. The bacteria has an average size of 350 nm (diameter) and is, therefore, impossible to see with a light microscope (Huang and Shah, 2023a). Thus, its small size and fastidious growth make it challenging to diagnose using traditional growth media and microscopy. Nowadays, diagnosis is made through polymerase chain reaction (PCR) assays targeting 16S ribosomal ribonucleic acid (rRNA), which is a pan-bacterial molecular diagnostic test (Cunliffe et al., 1996; Nelson et al., 1998).

*U. urealyticum* is an important cause of non-gonococcal urethritis (40 %–60 % of cases) (Huang and Shah, 2023a). Infections caused by this pathogen are typically uncomplicated and often asymptomatic. In certain instances, e.g., in the immunocompromised who are more susceptible to colonization of their mucous membranes by *Mycoplasma* and *Ureaplasma* species (Furr et al., 1994), it can be the cause of invasive infections such as osteomyelitis or infectious arthritis (Gremark and Axelsson, 2022).

In this article, we report on a case of a 26-year-old male with ocrelizumab-induced hypogammaglobulinemia who developed *U. urealyticum* osteomyelitis of the greater trochanter.

## 2 Case report

A 26-year-old male with a history of MS treated with ocrelizumab was referred to our tertiary center due to acute osteomyelitis of the left hip, likely linked to a complicated urinary tract infection with ongoing fever and positive urinalysis (pyuria, positive leukocyte esterase), despite antibiotics and consistently negative cultures.

Approximately 1.5 months prior, the patient was hospitalized twice in the urology department of the referring hospital due to recalcitrant urethritis with persistent fever and positive urinalysis despite empirical antimicrobial therapy with ciprofloxacin for 20 d. Multiple urine cultures were obtained before and during antibiotic therapy, but they all remained negative. Following the second hospitalization, fluconazole was added to the treatment regimen for 10 d after the detection of *Candida parapsilosis* in a urethral swab culture. Repeated urine and blood cultures all came back negative.

A total of 3 weeks after initial hospitalization, the patient's urological symptoms worsened, and his fever continued. A painful periurethral abscess developed that required drainage. Cultures remained negative; Gram staining revealed only the presence of white blood cells; and, subsequently, a urethrocutaneous fistula developed. A total of 2 weeks later, the patient presented with a painful left hip, persistent fever, and ongoing dysuria and hematuria. An ultrasound of the hip revealed joint effusion. This prompted the patient's transfer to the orthopedics department of the referring hospital, where a diagnostic hip arthroscopy was performed. Surprisingly, no aberrant findings were found during the procedure, and both tissue biopsy and fluid samples from the hip came back culture negative.

**Figure 1:**
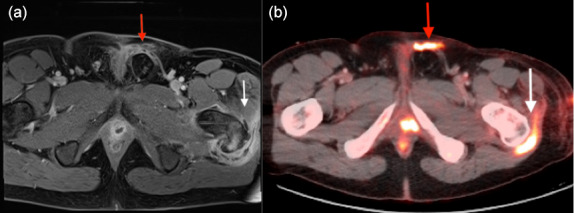
**(a)** MRI suggests osteomyelitis in the left greater trochanter (white arrow) and two small abscesses near the prostate with periurethral fluid infiltration (red arrow). **(b)** FDG PET/CT shows high metabolic activity at the trochanter (white arrow) and along the urethrocutaneous fistula's path (red arrow).

An MRI confirmed the presence of osteomyelitis in the greater trochanter (see Fig. 1a), leading to the patient's transfer to our tertiary orthopedic department. Throughout this period, the patient's inflammatory laboratory values remained consistently elevated.

Upon referral, a multidisciplinary team (MDT) meeting was called that included orthopedic surgeons, microbiologists, pharmacists, infectious disease specialist, and radiologists. The team decided to modify the patient's antimicrobial therapy to piperacillin–tazobactam and vancomycin administered intravenously. Next, an 18F-fluorodeoxyglucose positron emission tomography/computed tomography (FDG PET/CT) scan was carried out that identified increased metabolic activity in the greater trochanter region and another hypermetabolic area along the path of the urethrocutaneous fistula (Fig. 1b).

An open surgical debridement of the greater trochanter was performed to obtain tissue biopsies for cultivation. Additionally, broad-spectrum 16S rRNA PCR and amplicon sequencing (SepsiTest™-UMD; Molzym, Bremen, Germany) analysis was requested on a tissue biopsy. Secondary, micro-array analysis for detection of sexual transmitted diseases (STDs) was performed on a urethral swab. Over the next few weeks, the patient continued to experience fever and elevated inflammatory markers. As a result of ongoing purulent wound drainage, three additional surgical wound debridements were performed, yet all tissue biopsy cultures remained negative.

After 1 week, the STD panel showed a “weak positive” signal for *U. urealyticum*. After a 21 d period, the results of the 16S rRNA PCR assay returned positive for *U. urealyticum*. The prolonged analysis time was due to limitations of the Molzym 16S rRNA assay. The 16S rRNA PCR products were sequenced using sequencing primers (SeqGN16 and SeqGP16), supplied in the UMD Universal kit. However, it was stated in the manual that additional specific PCR assays are necessary for identification of *U. urealyticum*. Therefore, sequencing was repeated with modified primers to detect *U. urealyticum*. In response, vancomycin and piperacillin–tazobactam were replaced with doxycycline. Remarkably, within just a few days, all symptoms vanished, and inflammatory markers in the laboratory tests dropped significantly (normalization of white blood cell count and near-normalization of C-reactive protein within 5 d).

The patient was hospitalized for additional time due to dehiscence of the arthroscopy portal wounds. Luckily, this complication was promptly managed with negative-pressure wound therapy and localized wound care.

After 6 weeks of doxycycline treatment, the patient was symptom-free with normal hip exams and stable inflammatory markers. Further investigations suggest that ocrelizumab may have precipitated the rare infection by depleting B-cells and leading to hypogammaglobulinemia. Laboratory tests validated this, showing a complete lack of B-cells as evidenced by the absence of the CD19 marker on multiple blood samples. Due to the infection, ocrelizumab was discontinued, and intravenous immunoglobulin therapy started for the duration of B-lymphocyte depletion (ongoing at the time of writing).

## 3 Discussion

Ocrelizumab is a humanized monoclonal antibody targeting CD20 + B-cells that has been proven to reduce the frequency of relapse and disability progression in MS (Jhaveri and Lasalvia, 2019). Rituximab is an older and more available chimeric monoclonal antibody targeting CD20 + cells that is used for a variety of autoimmune disorders including MS (McGinley et al., 2017; Roos et al., 2023). B-cell-targeting medications, such as these agents, have been linked to serious infections via mechanisms such as prolonged B-cell depletion and hypogammaglobulinemia (Mikulska et al., 2018; Jhaveri and Lasalvia, 2019).

Patients with hypogammaglobulinemia, compared with immunocompetent individuals, are at increased risk of mucous membrane colonization by *Mycoplasma* and *Ureaplasma* spp., particularly in the urogenital tract. Increased colonization of mucosal membranes can escalate the likelihood of infection dissemination to distant sites, either through hematogenous or contiguous spread, potentially affecting bone and joints. This was shown in a study by Furr et al. (1994) that tracked 91 individuals with hypogammaglobulinemia. Out of these, 21 contracted infectious arthritis, with *Mycoplasma* and *Ureaplasma* spp. accounting for 38 % of the cases (Furr et al., 1994). In this case, we suspect hematogenous seeding of the greater trochanter resulting from the development and drainage of the periurethral abscess.

A 2019 literature review revealed a total of six cases in which invasive *Ureaplasma* infections were reported in patients who developed hypogammaglobulinemia during rituximab treatment (Jhaveri and Lasalvia, 2019). Our literature search revealed six other recent case reports of invasive *Ureaplasma* infections in patients treated with Rituximab (El Zein et al., 2023; Garzaro et al., 2022; Harold et al., 2021; Verhagen et al., 2021; Kvalvik et al., 2020; Poulsen et al., 2023). Multiple other invasive *Ureaplasma* infections were reported in patients with hypogammaglobulinemia due to other causes (Jhaveri and Lasalvia, 2019). We found only one other reported case of a severe *Ureaplasma* infection in a patient with hypogammaglobulinemia resulting from ocrelizumab treatment (Poulsen et al., 2023).

*Ureaplasma* infections often see diagnostic delays due to the organism's small size and complex growth requirements, evading traditional detection methods (Friberg, 1985). Specialized media or PCR is recommended for quicker identification. An MDT approach involving various specialties can expedite diagnosis and treatment. Thus, it is advisable to treat these patients in centers comprising multiple medical specialties (e.g., orthopedic surgeons, microbiologists, pharmacists, infectious disease specialist, and radiologists) experienced in the diagnosis and management of these rare cases (Walter et al., 2022).

*Ureaplasma* and other genital *Mycoplasma* species exhibit intrinsic resistance to β -lactam antibiotics and glycopeptides, as they lack a cell wall. This explains the lack of a therapeutic response after initiating vancomycin and piperacillin–tazobactam therapy. Furthermore, *Ureaplasma* spp. are naturally resistant to lincosamides except in high concentrations. Historically, *Ureaplasma* species have shown susceptibility to antibiotics belonging to the tetracycline, fluoroquinolone, and macrolide classes (Waites et al., 2005). Recent research indicates that antibiotic resistance to ciprofloxacin in *Ureaplasma* species is rising, reaching up to 59.8 %. On the other hand, resistance to other fluoroquinolones, such as levofloxacin and moxifloxacin, remains comparatively low, at 7.3 % and 5.3 %, respectively (Fernández et al., 2016; Wu et al., 2024). Meanwhile, resistance to macrolides and tetracyclines is still infrequent, with macrolide resistance at near 0 % and tetracycline resistance around 10 % (Fernández et al., 2016; Wen et al., 2023). Given the patient's prior ciprofloxacin treatment and the potential for fluoroquinolone resistance, the MDT chose to proceed with tetracycline therapy. The appropriate duration of antibiotic therapy for invasive *Ureaplasma* spp. infections is not well understood. The MDT decided on 6 weeks as is general practice for treatment of osteomyelitis.

## 4 Conclusion

Clinicians should be aware of the possibility of rare *Ureaplasma* or *Mycoplasma* infections in patients undergoing humoral immunosuppressive therapy, such as ocrelizumab or rituximab, in the presence of persistent urethritis despite broad-spectrum antimicrobial therapy. This population is at risk of more invasive infections, including osteomyelitis or infectious arthritis, particularly when the diagnosis is delayed. Early diagnosis can be established through specialized PCR assays, and consulting a microbiologist is recommended in such instances. A multidisciplinary approach is crucial for achieving an early diagnosis and developing an adequate treatment strategy.

## Data Availability

No data sets were used in this article.

## References

[bib1.bib1] Cunliffe NA, Fergusson S, Davidson F, Lyon A, Ross PW (1996). Comparison of culture with the polymerase chain reaction for detection of *Ureaplasma urealyticum* in endotracheal aspirates of preterm infants. J Med Microbiol.

[bib1.bib2] El Zein S, Garvey T, Amin S, Tande AJ (2023). Native joint polyarticular septic arthritis secondary to disseminated *Ureaplasma urealyticum*
infection in a patient on rituximab therapy with hypogammaglobulinemia: A Case Report. IDCases.

[bib1.bib3] Fernández J, Karau MJ, Cunningham SA, Greenwood-Quaintance KE, Patel R (2016). Antimicrobial susceptibility and clonality of clinical Ureaplasma isolates in the United States. Antimicrob Agents Chemother.

[bib1.bib4] Friberg J (1985). Diagnosis of genital Mycoplasma and Ureaplasma infections. J Reprod Med.

[bib1.bib5] Furr PM, Taylor-Robinson D, Webster AD (1994). Mycoplasmas and ureaplasmas in patients with hypogammaglobulinaemia and their role in arthritis: microbiological observations over twenty years. Ann Rheum Dis.

[bib1.bib6] Garzaro M, Zhao L-P, De Castro N, Mercier-Delarue S, Camelena F, Pereyre S, Gardette M, Berçot B, Malphettes M, Bébéar C, Bouaziz J-D, Le Goff J, Galicier L, Salmona M (2022). Metagenomic next-generation sequencing restores the diagnosis of a rare infectious complication of B cell depletion. Eur J Clin Microbiol.

[bib1.bib7] Gremark A, Axelsson O (2022). Urogenital Ureaplasma gav invasiv infektion hos immunsupprimerad [Urogenital Ureaplasma urealyticum can cause invasive infection in immunosuppressed patients]. Lakartidningen.

[bib1.bib8] Harold R, Simon GL, Akselrod H, Siegel MO, Roberts A (2021). *Ureaplasma* septic polyarthritis in a young woman with neuromyelitis optica receiving rituximab. BMJ Case Reports CP.

[bib1.bib9] Huang FS, Shah SS, Long SS (2023). Principles and Practice of Pediatric Infectious Diseases.

[bib1.bib10] Jhaveri VV, Lasalvia MT (2019). Invasive *Ureaplasma* Infection in Patients Receiving Rituximab and Other Humoral Immunodeficiencies – A Case Report and Review of the Literature. Open Forum Infect Dis.

[bib1.bib11] Kvalvik SA, Skarstein I, Veddeng A, Løland von Volkmann H, Kvalvik T, Torkildsen ØFG, Ebbing C (2020). An immunocompromised woman in her twenties with abdominal pain and vaginal discharge. Tidsskr Norske Laege.

[bib1.bib12] McGinley MP, Moss BP, Cohen JA (2017). Safety of monoclonal antibodies for the treatment of multiple sclerosis. Expert Opin Drug Saf.

[bib1.bib13] Mikulska M, Lanini S, Gudiol C, Drgona L, Ippolito G, Fernández-Ruiz M, Salzberger B (2018). ESCMID Study Group for Infections in Compromised Hosts (ESGICH) Consensus Document on the safety of targeted and biological therapies: an infectious diseases perspective (Agents targeting lymphoid cells surface antigens [I]: CD19, CD20 and CD52). Clin Microbiol Infec.

[bib1.bib14] Nelson S, Matlow A, Johnson G, Th'ng C, Dunn M, Quinn P (1998). Detection of *Ureaplasma urealyticum* in Endotracheal Tube Aspirates from Neonates by PCR. J Clin Microbiol.

[bib1.bib15] Poulsen EE, Jensen-Fangel S, Rudolf F (2023). Severe *Ureaplasma urealyticum* infection in a patient with ocrelizumab-induced hypogammaglobulinaemia. BMJ Case Reports CP.

[bib1.bib16] Roos I, Hughes S, McDonnell G, Malpas CB, Sharmin S, Boz C, Alroughani R, Ozakbas S, Buzzard K, Skibina O, van der Walt A, Butzkueven H, Lechner-Scott J, Kuhle J, Terzi M, Laureys G, Van Hijfte L, John N, Grammond P, Grand'Maison F, Soysal A, Jensen AV, Rasmussen PV, Svendsen KB, Barzinji I, Nielsen HH, Sejbæk T, Prakash S, Stilund MLM, Weglewski A, Issa NM, Kant M, Sellebjerg F, Gray O, Magyari M, Kalincik T, MSBase Study GroupDanish MS Registry Study Group (2023). Rituximab vs Ocrelizumab in Relapsing-Remitting Multiple Sclerosis. JAMA Neurol.

[bib1.bib17] Verhagen I, Oudenhoven H, van Welzen B, Kwok W-Y (2021). *Ureaplasma parvum* bacterial arthritis of the elbow in a patient with rheumatoid arthritis treated with rituximab. Rheumatology.

[bib1.bib18] Waites KB, Katz B, Schelonka RL (2005). Mycoplasmas and Ureaplasmas as Neonatal Pathogens. Clin Microbiol Rev.

[bib1.bib19] Walter N, Rupp M, Baertl S, Alt V (2022). The role of multidisciplinary teams in musculoskeletal infection. Bone Joint Res.

[bib1.bib20] Wen X, Nobakht MS, Yang Y, Kouhsari E, Hajilari S, Shakourzadeh MZ, Azizian K (2023). Tetracyclines resistance in *Mycoplasma* and *Ureaplasma* urogenital isolates derived from human: a systematic review and meta-analysis. 22.

[bib1.bib21] Wu Y, Majidzadeh N, Li Y, Zafar Shakourzadeh M, Hajilari S, Kouhsari E, Azizian K (2024). Trends of fluoroquinolones resistance in *Mycoplasma* and *Ureaplasma* urogenital isolates: Systematic review and meta-analysis. 36.

